# A Shepherd’s Crook Deformity of Proximal Femur Treated by Valgus Osteotomy and Bone Grafting

**DOI:** 10.7759/cureus.16485

**Published:** 2021-07-19

**Authors:** Subodh Kumar Pathak, Ajoy S. M., Praveen S Thivari, Atul Rai Sharma, Jasneet S Chawla, Aryan Sharma, Ashish Garg, Sarthak Sharma, Harish V. K. Ratna, Akshdeep Singh, Ayush Jain

**Affiliations:** 1 Department of Orthopedics, Maharishi Markandeshwar (Deemed to be University), Ambala, IND; 2 Department of Orthopedics, Ramaiah Medical College and Hospitals, Bangalore, IND; 3 Department of Orthopedics, Government Medical College and Hospital, Chandigarh, IND

**Keywords:** mccune-albright syndrome, fibrous dysplasia, allograft, dynamic hip screw, shepherd’s crook deformity

## Abstract

The Shepherd’s crook deformity of the proximal femur is a characteristic radiologic feature of fibrous dysplasia. It may be limited to a single bone, which is called monostotic, or may be polyostotic involving multiple bones as seen in McCune-Albright Syndrome. We report a case of a 19-year-old male patient who presented to us with pain in the right hip for one year. He had dysmorphic facies and multiple café-au-lait spots over the back, which were suggestive of McCune-Albright Syndrome. The radiographs of the hip showed varus deformity of the proximal femur. A lateral closing wedge osteotomy was done and the defect was filled with morselised femoral head allografts and fibular strut allografts. At the 14-month follow-up, the patient remained functionally active without any symptoms. The use of morselised femoral head allograft combined with strut fibular allograft ensures both stability and improved biology at the site of the lesion without any donor site morbidity.

## Introduction

Fibrous dysplasia is a rare benign developmental disorder of the bone, described by Lichtenstein as “gritty, greyish white fibrous tissue containing trabeculae of newly formed primitive bone”[1]. It is characterised by the replacement of the normal bone tissue by fibrous stroma and immature bone. These features may be limited to a single bone which is called monostotic or may be polyostotic involving multiple bones as seen in McCune-Albright Syndrome. The varus deformation (shepherd’s crook deformity) of the proximal femur is a characteristic radiologic feature of fibrous dysplasia. The fibro-osseous tissue at the replaced bone matrix is weak, resulting in soft bones, which sustain micro-fractures and undergo progressive bowing and varus angulation under physiological loading of the bones. The natural course of such deformity in the proximal femur in the absence of any intervention results in pathological fractures. The ideal management of deformities remains a matter of debate. The goals of the treatment are to relieve symptoms and correct the deformity and provide mechanical support for the weakened bones. A variety of surgical methods like curettage and bone grafting, internal fixation with either plates or intramedullary nails, Ilizarov fixation have been reported with mixed results [2]. Here, we report a 19-year-old male patient with polyostotic fibrous dysplasia with shepherd’s crook deformity of the hip, which was managed with dynamic hip screw, fibular strut allograft and cancellous allograft, with a favourable outcome.

## Case presentation

**Figure 1:**
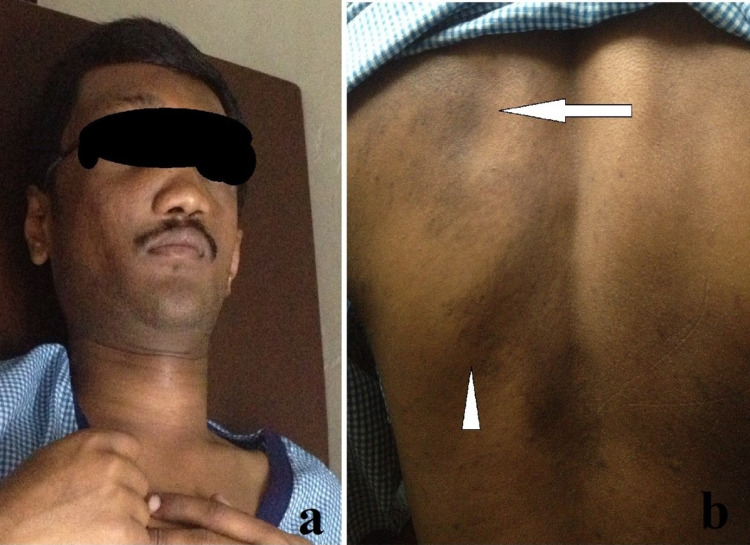
(a) Dysmorphic features seen over the face of the patient. (b) Multiple hyperpigmented macules with irregular margins (café-au-lait) spots over the posterior aspect of the trunk (white arrow and white arrowhead).

A 19-year-old male patient had presented to our outpatient department with pain in the right hip and thigh which was of gradual onset, increasing in intensity over a period of 12 months which had worsened over the last three to four months before presenting to us. The clinical examination of the patient had revealed dysmorphic facies with multiple hyperpigmented macules with irregular margins (café-au-lait spots) noticed over the posterior aspect of the trunk (Figures 1a, 1b).

**Figure 2:**
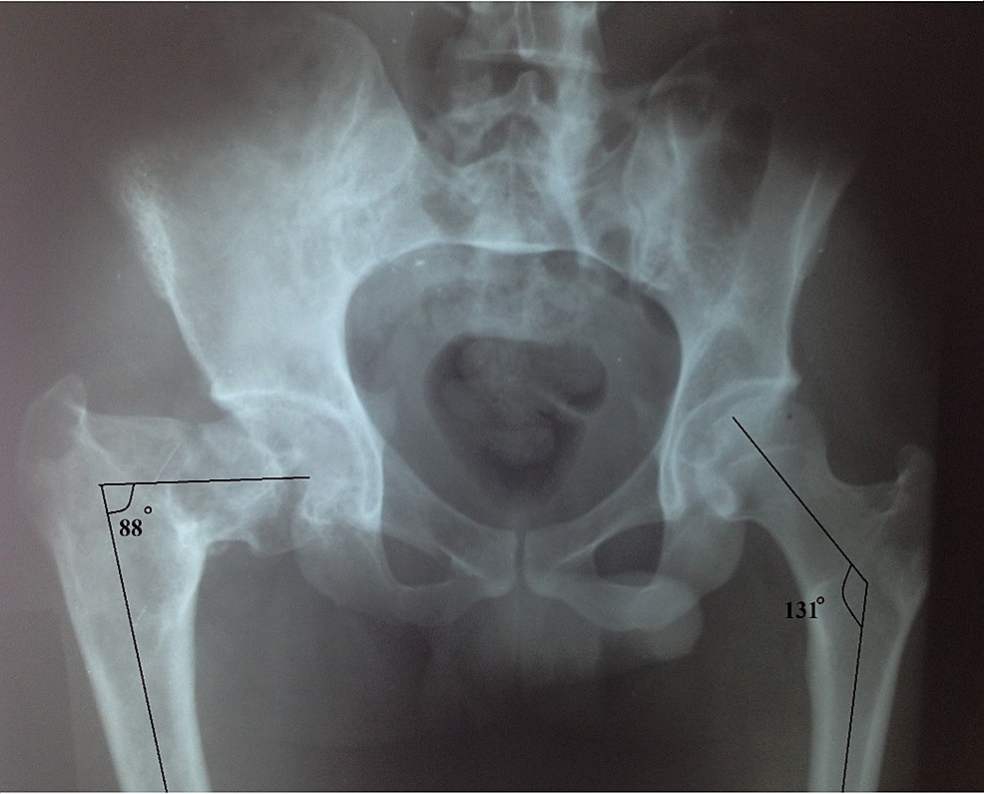
Anterior-posterior radiograph of pelvis showing right pre-operative neck-shaft angle as 88.0 degrees.

Tenderness was noted over the hip joint and around the thigh and range of motion at the left hip was restricted with flexion of 0 to 100 degrees, abduction of 0 to 30 degrees, adduction of 0 to 20 degrees, internal rotation of 0 to 20 degrees and external rotation of 0 to 40 degrees. There was a shortening of the right lower limb by 3 cm. Plain radiographs of the hip showed a varus deformity of the proximal femur with the neck-shaft angle of 88.0 degrees and loss of trabecular pattern with multiple radiolucent ground-glass opacities in the proximal femur were suggestive of fibrous dysplasia (Figure 2).

The appendicular skeleton and skull were screened for the presence of the asymptomatic lesions which showed a lesion of the left humerus. The clinicoradiological features were suggestive of McCune-Albright Syndrome with polyostotic fibrous dysplasia. Baseline blood investigations performed were within the normal range. The serum alkaline phosphatase was raised at 252 IU/L (50-136 IU/L). We ran an endocrine profile, including thyroid and parathyroid function, adrenal function, serum prolactin, and sex hormone levels. The thyroid profile was suggestive of hyperthyroidism. The patient was advised surgical correction of the deformity and fixation.

**Figure 3:**
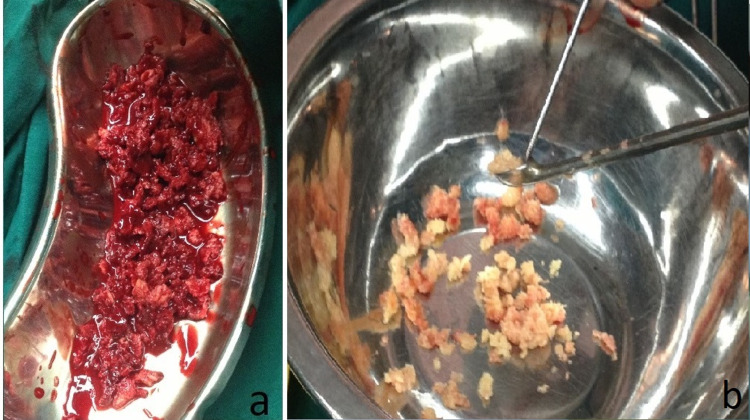
(a) Collected fibrous tissue sample from the lesion sent for histopathology. (b) Morselised femoral head allografts harvested from a bone bank.

The plan for surgery involved preparing the paper templates of the deformity by tracing it onto a sheet of white paper from the radiographic films. A lateral closing wedge osteotomy was planned. The patient was put in the supine position on a fracture table. A lateral incision of appropriate length centred over the greater trochanter was used to access the proximal femur. The osteotomy was performed as planned at the level of the lesser trochanter. The lesions were completely curetted and the tissue samples were collected for histopathological examination (Figure 3a). The bone defects so created were filled with morselised femoral head allografts harvested from the bone bank (Figure 3b).

**Figure 4:**
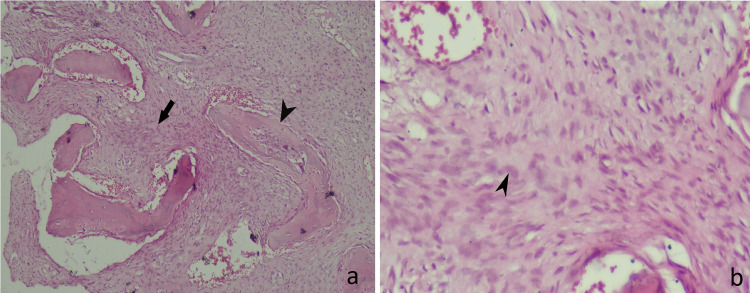
(a) Curvilinear trabeculae of woven bone (black arrowhead) in the fibrous background with no osteoblastic cells (black arrow). (b) Cytologically bland stromal spindle cells (black arrowhead).

A fibular strut allograft was also incorporated into the lesion. The osteotomy was stabilised with a dynamic hip screw. The final neck-shaft angle achieved was 133 degrees. The biopsy showed curvilinear trabeculae of woven bone in the fibrous background with no osteoblastic rimming. There were spindle cells present in the stroma which were cytologically bland (Figures 4a, 4b).

**Figure 5:**
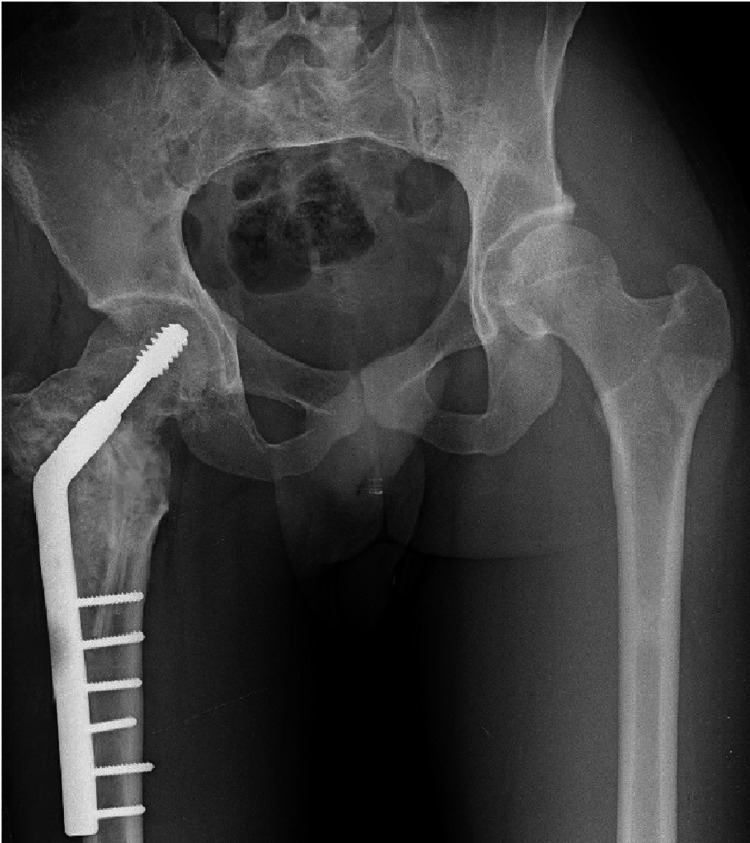
Antero-posterior radiograph showing valgus neck-shaft angle and healing of the lesion at 14-month follow-up.

The patient was allowed to ambulate with protected weight-bearing after eight weeks. The patient was followed up in the outpatient department at regular intervals of six weeks, three months, six months and one year. At six months follow-up, there were signs of bony union and graft integration on radiographs. Functional performance improved with time and the patient could perform his regular activities by 6 months after surgery. At 14 months follow-up, the range of motion showed improvement with painless passive flexion of 0 to 130 degrees, abduction of 0 to 40 degrees, adduction of 0 to 30 degrees and internal rotation of 0 to 30 degrees. The patient remained functionally active without any clinical or radiological recurrence (Figure 5).

## Discussion

The management of fibrous dysplasia remains a challenge to the treating surgeon. The condition most commonly affects the long bones, which are weakened and are at risk of deformity. The proximal femur is especially at risk for deformation due to weight-bearing resulting in progressive bowing and varus deformity, classically called the Shepherd’s Crook deformity. The resulting deformity may manifest as limp in patients due to limb-length discrepancy. In progressive disease, it may cause painful hip and ultimately pathological fractures.

The treatment of the deformity is individualised and is guided by the patient’s age, symptoms, the severity of deformity and the presence of associated pathological fractures. Severe deformity with painful hip usually requires surgical correction with careful preoperative planning with templates to identify the level and amount of correction required. Commonly, corrective medial displacement subtrochanteric valgus osteotomy with bone grafting has been recommended. The implant of choice to obtain adequate fixation to maintain the correction achieve good stability remains a matter of debate. Some authors have preferred intramedullary devices whereas others have described the use of extramedullary implants. Yang et al. had performed the valgus osteotomy followed by intramedullary nailing and impaction bone grafting with successful correction of the deformity without any pathological fractures or recurrence of the deformity at average 75.3 months of follow-up [3]. Hefti et al. used a novel intramedullary nail that could be inserted through the site of osteotomy and passed in retrograde fashion in to neck after achieving an adequate neck-shaft angle. They had reported satisfactory results in a case series of 15 patients where most of them were pain-free and could ambulate well [4]. However, O’Sullivan pointed out that the neck-shaft angle tends to decrease in the long term with intramedullary nailing [5].

In our case, we relied on an extramedullary implant, a dynamic hip screw, to maintain the neck-shaft angle and obtain a stable reduction. Many of the studies have reported the successful use of this implant after valgus osteotomy [6,7]. The success of these implants relies on the fixation stability of the implant, which can be achieved with a long side plate. The critics of the implant point to the difficulty in contouring the plate to the deformed shaft of the femur. But we did not find any such difficulty in our case. The implant was stable during the entire course of follow-up. Some authors have also reported the use of Ilizarov fixator in the proximal femur which has been shown to enable early weight-bearing and excellent leg functions.

Bone grafts play an important role in healing the lesion and preventing recurrence. However, the type of bone graft, the quantity, and the source remain a matter of choice. The earlier attempts at using cancellous bone graft after curettage of the lesion have been shown to have had early resorption and recurrence of lesions [8]. The cortical fibular strut graft has been considered a suitable alternative that can provide much-needed mechanical stability [9]. However, few authors have reported no added advantage of using either the cortical or cancellous graft in bone healing [10]. However, Yang et al. had performed impaction grafting of the lesion and reported successful union without any recurrence of deformity in their case series involving 13 patients over four years [3]. In our case, we had used processed allografts obtained from our institutional bone bank. A morselised femoral head cancellous graft and a fibular strut graft were harvested from the bone bank. The use of allografts had the advantage of ensuring the availability of both cortical and cancellous graft in adequate quantity at the graft site, which is usually not possible with autografts. It also reduced the morbidity of the patient as multiple sites would have to be used to harvest adequate graft material. Though our technique did not emphasize impacting the graft into the lesion, the femur achieved a successful union without graft resorption or recurrence of the deformity at the end of 14 months follow-up. It is evident from this case that, in carefully selected symptomatic patients with proximal femur deformity of fibrous dysplasia, surgical correction of deformity to align the mechanical axis and anatomical axis with the addition of abundant allograft can be a better option.

## Conclusions

The optimal management option for fibrous dysplasia of the proximal femur remains debatable. Correction of the varus deformity and internal fixation with dynamic hip screw has a satisfactory functional outcome, which prevents recurrence and pathological fractures. The use of morselised femoral head allograft combined with strut fibular allograft ensures both stability and improved biology at the site of the lesion without any donor site morbidity.
